# T11. CEREBROSPINAL FLUID (CSF) MARKERS OF INFLAMMATION AND INFECTIONS IN SCHIZOPHRENIA AND AFFECTIVE DISORDERS: A SYSTEMATIC REVIEW AND META-ANALYSIS

**DOI:** 10.1093/schbul/sby016.287

**Published:** 2018-04-01

**Authors:** Sonja Orlovska, Ole Köhler-Forsberg, Sophie Brix, Merete Nordentoft, Daniel Kondziella, Jesper Krogh, Michael Benros

**Affiliations:** 1 Mental Health Centre Copenhagen, Copenhagen University Hospital; 2 Copenhagen University Hospital; 3 Mental Health Centre Copenhagen

## Abstract

**Background:**

Infections and inflammatory processes have been associated with the development of schizophrenia and affective disorders; however, no study has yet synthesized all the available data on cerebrospinal fluid (CSF) immune-alterations. We conducted the first systematic review of the immunological findings from CSF studies among patients with schizophrenia or affective disorders.

**Methods:**

All studies investigating CSF inflammatory markers in persons with schizophrenia or affective disorders published prior to March 23, 2017 were identified searching PubMed, CENTRAL, EMBASE, Psychinfo, and LILACS. The literature search, data extraction and assessment of risk of bias were performed by two independent reviewers. Meta-analyses with standardized mean difference (SMD) including 95% confidence intervals (CI) were performed on studies including healthy controls.

**Results:**

We identified 112 CSF studies published between 1942–2016, of which 32 were included in meta-analyses; however, only few studies investigated identical biomarkers. The CSF/serum albumin ratio was increased in both schizophrenia (54 patients; SMD=0.62; 95%CI=0.24–1.00) and affective disorders (302 patients; SMD=0.43; 95%CI=0.25–0.61, I2=0%), compared to healthy controls. Total CSF protein was elevated in both schizophrenia (97 patients; SMD=0.38; 95%CI=0.12–0.65, I2=0%) and affective disorders (53 patients; SMD=0.77; 95%CI=0.36–1.18, I2=0%). The IgG ratio was increased in schizophrenia (54 patients; SMD=0.60; 95%CI=0.23–0.98), whereas the IgG Albumin ratio was decreased (32 patients; SMD= -0.62; 95%CI= -1.13 to -0.12). Interleukin-8 (IL-8) (95 patients; SMD=0.46; 95%CI=0.17–0.75, I2=0%) and IL-6 levels (230 patients; SMD=0.38; 95%CI=0.02–0.74; I2=64%) were increased among individuals with schizophrenia but not significantly increased in affective disorders. None of the remaining inflammatory markers were significantly different compared to healthy controls in the meta-analyses. However, in the studies which did not include healthy controls, CSF abnormalities were more common, and CSF dependent re-diagnosis occurred in 3.2–6% in the two studies investigating this.

**Discussion:**

Our findings suggests that schizophrenia spectrum and affective disorders are associated with CSF abnormalities including signs of blood-brain barrier impairment and inflammation, supporting a role of the immune system in mental disorders. However, only few studies investigated the same parameters with healthy controls and high quality longitudinal CSF studies are lacking, including impact of psychotropic medications and potential benefits of anti-inflammatory treatment in subgroups with abnormal CSF inflammatory markers

